# Exploring the utility of the 3-minute all-out running test in assessing match performance and external loads in university soccer players

**DOI:** 10.1371/journal.pone.0323655

**Published:** 2025-06-02

**Authors:** Duncan Peter Sutcliffe, Mark Kramer

**Affiliations:** Physical Activity, Sport and Recreation (PhASRec), North-West University, Potchefstroom, Potchefstroom Campus, South Africa; eCampus University: Universita degli Studi eCampus, ITALY

## Abstract

**Purpose:**

This study evaluated the utility of the 3-minute all-out running test (3MT) in assessing match performance and physiologically relevant load metrics among male university soccer players. We aimed to explore the relationship between 3MT metrics, including critical speed (CS), finite energy reserve (D’), and maximal running speed (S_max_), with key match performance indicators, while also assessing the efficacy of a modified method for tracking player load.

**Methods:**

Twenty-six male university soccer players completed the 3MT to determine CS, D’, and maximal speed (S_max_). Match performance data (n = 3–8 matches per player) were concurrently gathered, including total distance covered, high-speed running (HSR), very high-speed running (VHSR), and overall player load. Correlation analyses were conducted to examine the relationships between 3MT variables and match performance, with a specific focus on how a modified load-tracking method compared to traditional approaches.

**Results:**

Players with higher CS exhibited smaller fluctuations in D’ kinetics, indicating more efficient energy utilization during matches. A strong positive correlation was found between 3MT-derived S_max_ and the initiation of both HSR (r = 0.73, *p* < 0.01) and VHSR (r = 0.69, *p* < 0.05), highlighting the link between sprint capacity and match-specific high-intensity efforts. Lastly, a modified method for assessing player load demonstrated a high correlation with traditional methods (r = 0.99), while also accounting for individual metabolic demands. The modified approach offers a more nuanced understanding of player workload, potentially improving fatigue management, performance, and injury prevention.

**Conclusions:**

The 3MT proved to be a valuable tool for evaluating individual physiological conditioning while also mapping on to key match performance metrics and external loads in university soccer players. The association with key performance indicators such as high-speed running capacity and sustained effort highlights its potential for optimizing training strategies.

## Introduction

Soccer matches incorporate repeated bouts of high-intensity running (HIR) coupled with prolonged periods of low-intensity running (LIR) and walking [1–3]. Conventional parameters that reflect the physical performances of soccer are frequently classified according to the running intensity of players which is predominantly based on the speed of movement This data is derived from time-motion analyses (TMA) which is a standard evaluation tool that uses global positioning systems (GPS) to track distances and speeds covered during matches [4]. Although attempts have been made to quantify the physical loads on players during matches using TMA-based metrics, these metrics are often based on fixed thresholds rather than on thresholds supported by more robust physiological underpinnings [5]. The critical speed (CS) concept offers a potential solution given that the CS metric operates as an indicator of the critical metabolic rate and as an external workload that delineates sustainable from unsustainable exercise [6]. Subsequently, there is a limited understanding of how conventional match parameters relate to those based on the CS concept especially during match play.

It is agreed that the metrics representative of external workloads (e.g., total distance covered during a match, distance covered during HIR and number of HIR’s) vary between playing positions [7–9], as midfielders consistently cover greater distances during HIR compared to the other playing positions [10,11]. A limitation of the external workload classification of current GPS-based metrics extends beyond the arbitrary values used to define speed thresholds, in that there are inconsistent classifications both between- and within sporting codes [5]. If the purpose is to monitor the work capacity of each player, or identify the magnitude of fatigue at the level of the individual, the use of personalised thresholds derived from physiologically relevant criteria, such as CS, would likely be more suitable compared to conventional match parameters [12]. Moreover, whether the relative time and distance covered above or below speed thresholds, including CS, during match-play may be more informative and closely linked to individual fatigue and/or performance mechanisms in comparison to conventional match play parameters would require further exploration and represents a gap in the current literature.

In terms of external workload and fatigue monitoring, comparisons with conventional methods (e.g., distance covered at HSR) in relation to the CS concept have not been previously explored [13]. Critical speed assessments yield two key parameters, namely CS which is representative of aerobic capacity, and D’ which is representative of finite energy reserves for covering distances at speeds above CS [6,14,15]. These parameters (i.e., CS, D’) can be readily derived from a 3-minute all-out running test (3MT), presenting a time-efficient method for obtaining key metrics for team-sport athletes [16]. Recent advancements in our understanding of D’ and D’-kinetics have permitted the rudimentary monitoring of D’ depletion and recovery during track running [17,18], but have not been implemented using high-fidelity GPS technologies nor applied to field-sports such as soccer. Whether the proposed method of analysing D’ kinetics may potentially provide more insightful information on the fatigue and/or performance responses as well as match-loads of players during soccer matches has not been previously examined and therefore presents a novel gap in the literature. Given that approximations of player load are important for decision-making purposes such as recovery strategies, it remains to be explored whether CS parameters can be used to approximate or inform player load compared to more traditional metrics.

The present study therefore sought to explore the following objectives: (i) to assess the relationships between the variables from a 3MT and traditional match performance variables in university level soccer players, (ii) to evaluate whether CS and D’ can provide insights into match performances by tracing the D’-kinetics across multiple matches, and (iii) whether 3MT-based metrics can provide analogous indicators of match-load compared to more traditional metrics.

## Materials and methods

### Participants

Twenty-six (n = 26) male Division-I university soccer players (age: 21.36 ± 2.58 years; body mass: 62.04 ± 7.47 kg) volunteered for the study. The sample size was determined *a priori* using a paired t-test design, considering the following factors: (i) an effect size (d_z_) of 0.5, (ii) a type-1 error rate of 5%, and (iii) a desired power of 80%, resulting in a type-2 error rate of 20%, and (iv) a one-sided tail. Participants received both verbal and written explanations of the study. An information session was held at the soccer club, during which interested players were provided with a written informed consent form. They were given 72 hours to review and sign the form, contingent upon meeting the inclusion criteria. Eligible participants had to be male soccer players from the university’s Soccer Institute, aged 18–25 years, of any ethnicity or race to ensure inclusive ethical practices, injury-free, and not engaged in strenuous training 24–48 hours prior to testing. All participants were competing in the ABC Motsepe Regional League. Exclusion criteria consisted of failure to provide informed consent and current injuries (i.e., < 6 months) or illnesses affecting performance. This study was approved by the Health Science Ethics Committee (HREC) of North-West University (NWU-00032–22-A1) and conducted in accordance with the Declaration of Helsinki [19].

### Design

This research article utilised a selected-group, observational, cross-sectional design. Participants took part in three separate data collection phases: (1) familiarisation with all testing procedures, (2) the 3MT to determine CS, D’, and maximal sprint speed attained during the 3MT (S_max_) and (3) TMA of 3–8 matches per player to aggregate performances during competitive league matches. The order of testing was not randomised since there was a potential of potentiation that may influence performances. All data collection was conducted by a qualified sport scientist with expertise in performance analysis and player monitoring, ensuring standardised testing procedures and reliable data acquisition.

### Methodology

#### Three‑minute all‑out running test (3MT).

Before starting the 3MT testing, participants engaged in a comprehensive warm-up routine that included jogging, sprints and dynamic stretching, followed by a 5-minute rest period [20]. Thereafter each participant was equipped with a global positioning system (GPS) unit that sampled data at 10 Hz (MinimaxX S4 V4.0, Catapult Innovations, Victoria, Australia). The GPS units were secured to the upper back of each participant via a customised vest and was activated 10 minutes prior the start of the 3MT to ensure optimal satellite connectivity. For the actual 3MT, participants were instructed to run at maximum effort for the entire 3-minutes around two soccer standardised, grass, soccer fields while wearing their soccer boots to more closely simulate match-play conditions. Continuous verbal encouragement was provided to maintain maximal effort, but participants were not given any timing information to avoid potential influences of pacing. After the 3MT, data were extracted and exported to Matlab (R2022a, Mathworks™, Natick, MA, USA) to analyse parameters such as maximal sprint speed (S_max_), CS, and D’ values [21]. The GPS system used is known for its validity and reliability in measuring instantaneous velocities (r = 0.96), accelerations (r = 0.98), and decelerations (r = 0.98) during team sports running [22]. Additionally, the 3MT has been validated for its reliability, showing high intraclass correlation coefficients (ICC) between two trials: r = 0.95 for D’ and r = 0.96 for CS [23]. Prior to all testing, participants were provided with the following instructions: (i) refrain from vigorous exercise 24–48 hrs prior, (ii) avoid caffeine intake 4 hr prior, (iii) avoid alcohol consumption 24 hrs prior, (iv) arrive in a well hydrated state and be ~ 2 hrs post-prandial.

#### Match Performances.

Testing took place during the representative university soccer season. A total of 26 participants from the institutional soccer teams participated in 3–8 soccer matches with goalkeepers excluded from the analysis. Match performance data were recorded during these competitive league matches which took place from the 5^th^ of February to the 3^rd^ of March during the 2023 season. The participants played two matches each week, on Thursdays and Saturdays. All matches were held on a standardised, grass soccer field and consisted of two 45-minute halves. All data were normalized per minute played to account for differences in total playing time and match stoppages [24]. Prior to match play, participants were fitted with GPS units sampling at 10 Hz (MinimaxX S4 V4.0, Catapult Innovations, Victoria, Australia). The GPS units were attached to the upper back of each participant using a harness and were activated 10 minutes before each match. For a movement to be recorded as an effort, players had to maintain a specific velocity for at least 0.5 seconds [24]. An intelligent motion filter incorporated within the GPS unit and software was used to exclude non-game activity by considering stoppages in play, such as during half-time and at the end of play. The raw data was further divided into first-half and second-half files. GPS Doppler data was used during the analysis of the GPS-related variables. All raw match GPS datafiles were extracted and analysed using custom scripts (Matlab (R2022a, Mathworks™, Natick, MA, USA).

The 3MT parameters for each player were used to evaluate the *modified* match performance parameters. All *conventional* and *modified* match performance parameters are represented in Table 1

**Table 1:** The *conventional* and *modified* match performance parameters and their corresponding definitions.

Abbreviation	Conventional Match Parameters Definition
D_ < LSR_	Distance covered during low-speed running (LSR, < 4 m/s)
D_ > HSR_	Distance covered during high-speed running (HSR, > 4 m/s)
D_>VHSR_	Distance covered during very high-speed running (VHSR, > 5.5 m/s)
Initiate_HSR_	Number of times a speed above HSR was initiated
Initiate_VHSR_	Number of times a speed above VHSR was initiated
D_Total_	Mean total distance covered
Initiate_MatchSmax_	Number of times speed exceeds 90% of S_max_ determined during the match
**Abbreviation**	**CS-based Match Parameters Definition**
Time>CS	Time spent at speeds above CS
Initiate_CS_	Number of times a speed above CS was initiated
Initiate_Smax_	Number of times speeds exceeds 90% of S_max_ determined from the 3MT
D_ > CS_	Distance covered above CS
D_ < CS_	Distance covered below CS
ΔD’_bal_	Change in D’_bal_ from maximal to minimal values achieved during a game
D’_bal_ _Diff_	Difference in D’_bal_ between maximal and minimal values achieved during the match
D’_bal End_	D’_bal_ remaining at the end of the match
D_>Smax_	Distance covered at speeds above 90% of S_max_ from the 3MT

*Note: conventional match parameters are derived directly from the match itself, while CS-based match parameters are obtained by identifying 3MT variables and integrating them into the match analysis.*

#### Player Load.

Monitoring player load during a match is crucial for managing athlete fatigue, reducing injury risks and optimising player performances [25]. The traditional methods of assessing player load are represented as Load_traditional_ (Equation 1). While these metrics provide valuable information, they may not fully capture the nuanced physiological demands experienced by players across different intensity zones. To address this, we propose a modified player load metric, Load_modified_ (Equation 2), which focuses on parameters that offer unique insights into the player’s physiological responses. We have not included metrics that would be common in both player load calculations (e.g., accelerations, total distance etc) as we only wanted to focus on those parameters that would provide unique insights. As such, the Load_modified_ metric would encapsulate distances covered within the moderate-, heavy-, and severe-intensity zones, which have robust physiological underpinnings compared to metrics that form part of the Load_traditional_ metric. The traditional external player loads achieved during a match was evaluated as follows:


$$\text{Loa}{\text{d}}_{\text{traditional}}\text{= }{\text{D}}_{\text{LSR}}\text{+ }{\text{D}}_{\text{HSR}}\text{+ }{\text{D}}_{\text{VHSR}}\text{+ Initiat}{\text{e}}_{\text{HSR}}\text{+ Initiat}{\text{e}}_{\text{VHSR}}\text{+ Initiat}{\text{e}}_{\text{Smax}}$$ [Equation 1]

Where D_LSR_ is distance covered while engaged in low-speed running; D_HSR_ is distance covered while high-speed running; D_VHSR_ is distance covered while engaged in very high-speed running; Initiate_HSR_ is the number of times HSR was initiated; Initiate_VHSR_ is the number of times VHSR was initiated; and Initiate_Smaz_ is the number of times 90% of maximal sprinting speed was initiated.

The modified external player loads achieved during a match was evaluated as follows:


$$\text{Loa}{\text{d}}_{\text{modified}}={\text{D}}_{<\text{CS}}+{\text{D}}_{\text{CS}}+{\text{D}}_{\text{Smax}}+\text{Initiat}{\text{e}}_{\text{CS}}+\text{Initiat}{\text{e}}_{\text{Smax}}+△\text{D}{\;\;\;\prime \;\;\;}_{\text{bal}}$$ [Equation 2]

Where D_<CS_ is distance covered below critical speed (i.e., moderate-intensity zone); D_CS_ is distance covered above critical speed (i.e., heavy-intensity zone); D_Smax_ is distance covered at 90% of S_max_ achieved during the 3MT; Initiate_CS_ is the number of times speeds above CS were initiated; Initiate_Smaz_ is the number of times 90% of maximal sprinting speed achieved during the 3MT was initiated; and ΔD’_bal_ is the greatest D’-depletion experienced during match-play and therefore represent the maximal sustained efforts within or beyond the heavy-intensity domain.

All load values were divided by 1000 for better visualisation and interpretation.

### Statistical Analyses

All statistical analyses were completed using the R-programming language (version 2023.06.1) [26]. Data were evaluated for normality using the Shapiro-Wilk test where deviations from normality were accepted at *p* < 0.05. All data are presented as mean ± standard deviation (SD) unless stated otherwise.

The associations between CS-based parameters and traditional match parameters were evaluated using the Pearson correlation coefficient. The magnitudes of the correlation coefficients were interpreted as follows: negligible: 0.00–0.10; weak: 0.10–0.39; moderate: 0.40–0.69; strong: 0.70–0.89; very strong: 0.90–1.00 [27]. To evaluate the difference between traditional and modified player load metrics a paired t-test was used. Hedge’s g served as a measure of the standardised effect size which was interpreted as follows: trivial: < 0.20; small: -0.20–0.59; moderate: 0.60–1.20; large: > 1.20.

## Results

**Fig 1:**
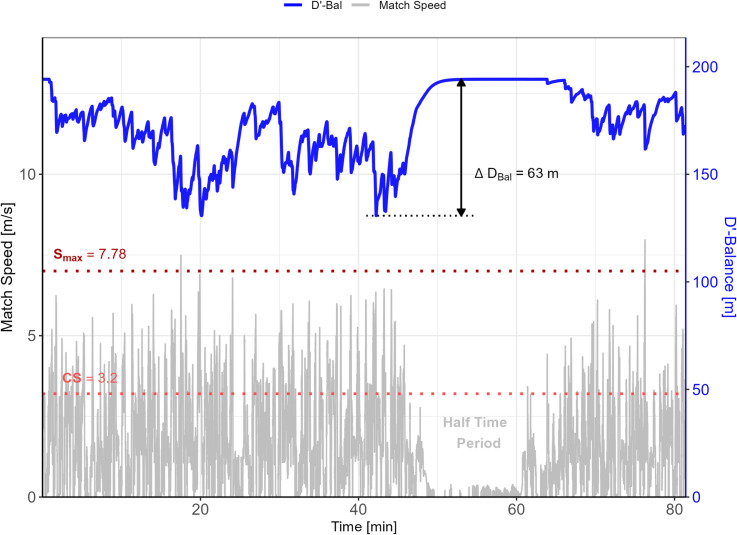
Match data from a representative participant. Note: The instantaneous running speed (grey line) is shown for the full match played. For each instance when running speeds exceed CS (light red horizontal line), a portion of D’ is depleted (blue line) which tends to recover as soon as running speed is below CS. Dark red horizontal line represents 90% of maximal speed (S_max_) achieved during a 3MT.

The match data from a representative participant are presented in Fig 1 which highlights the variations in running speeds and the corresponding impact on the player’s finite energy reserves.

**Fig 2:**
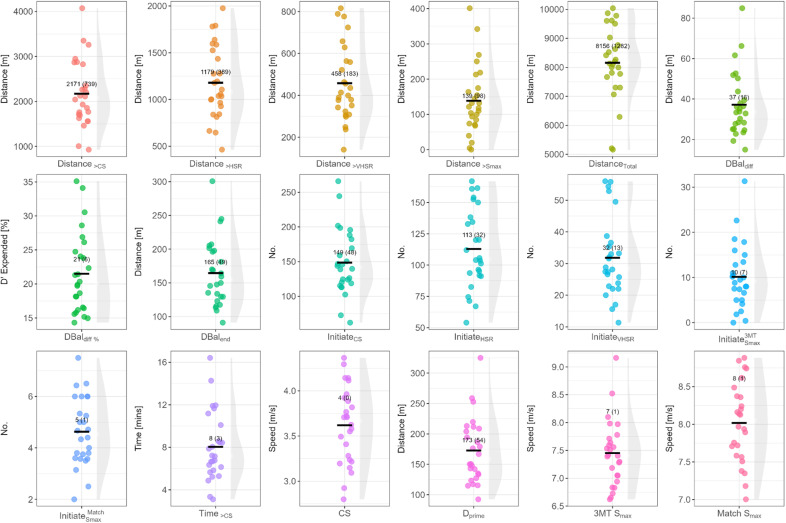
Descriptive statistics for 3MT and match data. Note: black horizontal bar indicates the mean value; shaded grey areas indicate the underlying distribution of the data points; HSR = high speed running (>4 m/s); VHSR = very high-speed running (>5.5 m/s); Dbal = D’ balance; DBal Diff = D’ balance difference; CS = critical speed; S_max_ = maximal speed attained during 3MT.

The data for each participant and descriptive statistics (mean, SD) for each of the variables of interest are presented in Fig 2. The individual data points show the variability in performance within a team.

**Fig 3:**
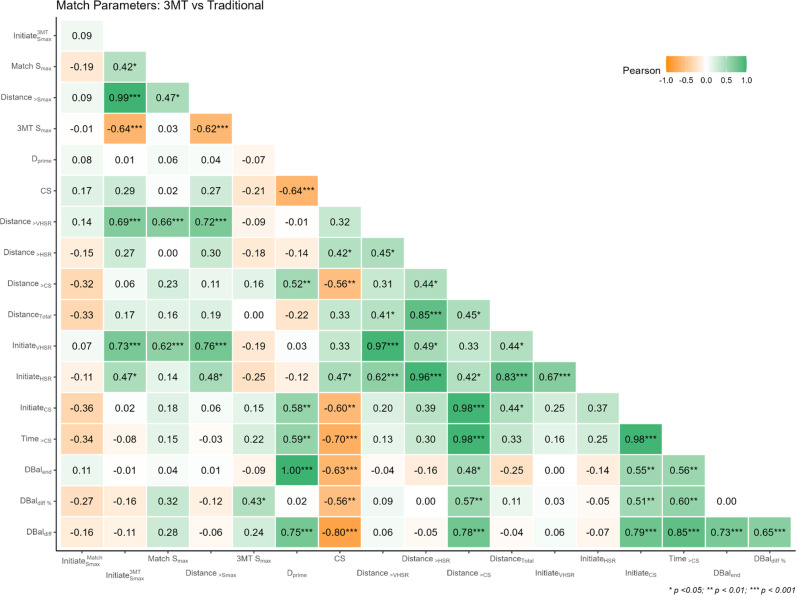
Correlation analysis between 3MT and match parameters. Note: HSR = high speed running (>4 m/s); VHSR = very high-speed running (>5.5 m/s); Dbal = D’ balance; DBal Diff = D’ balance difference; CS = critical speed. The magnitude (opacity) and direction of the correlations (colour) are indicated by the colour scheme (green = positive; orange = negative).

The results for the correlation analyses between 3MT and match parameters are shown in Fig 3, highlighting the association between physiological and match performance metrics.

**Fig 4:**
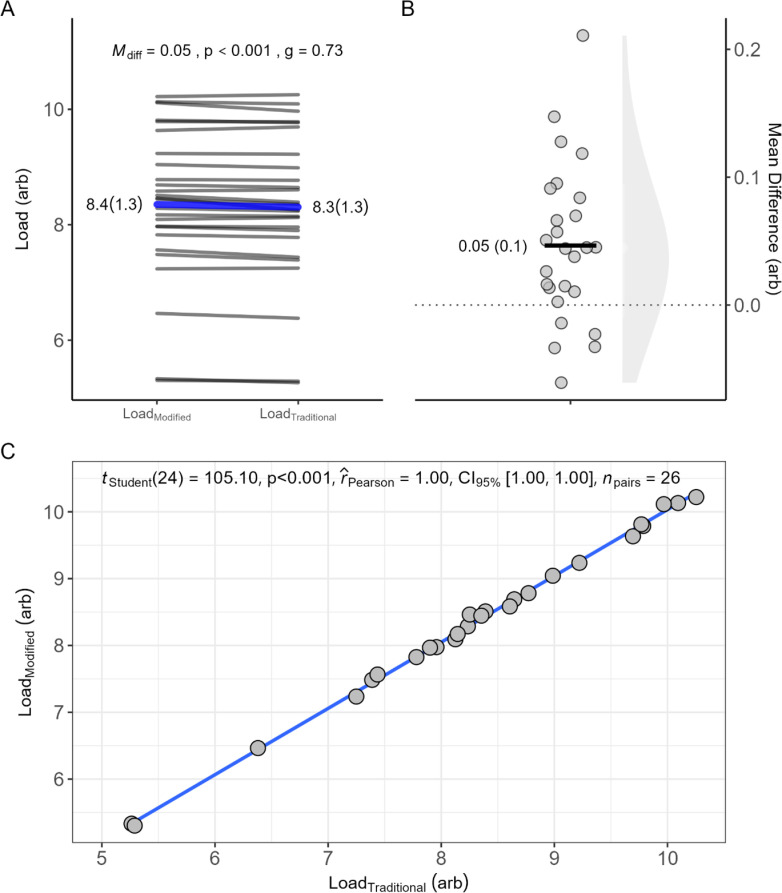
Player load comparison from the traditional metrics compared to CS-based metrics. Panel A shows the paired comparisons between the traditional and modified methods of classifying player loads; panel B shows mean difference between the modified and traditional played load scores; panel C highlights the relationship between the traditional and modified methods of classifying player loads. *Note: M*_*diff*_* = mean difference; r*_*Pearson*_* = Pearson correlation coefficient; g = Hedge’s g.*

The results of the player load comparison are presented in Fig 4. Both the traditional and modified methods demonstrate a remarkably strong association (r = 0.99), indicating that they consistently measure player load in a similar manner. Despite this high level of correlation, the analysis reveals statistically significant differences of 0.05 units between the two methods. This finding underscores the reliability of both approaches in assessing player load while highlighting the necessity for careful consideration of minor discrepancies when making decisions related to player monitoring and performance evaluation.

## Discussion

Our study sought to investigate the utility of 3MT-related variables within the context of match performance analysis and assess the efficacy of alternative methods for tracking player load. We identified three novel findings: (i) higher CS values were associated with reduced fluctuations in D’_bal_-_Diff_, suggesting more efficient energy utilization; (ii) moderate-to-strong, positive correlations were found between 3MT-based metrics and traditional metrics, emphasizing the more physiologically justifiable nature of such metrics to evaluate soccer performances; and (iii) a *modified* method for measuring player load demonstrated a high correlation with the *traditional* load while accounting for individual metabolic demands.

Regarding our first finding, CS exhibited a strong negative correlation with D’_bal-Diff_ (r = -0.80, *p* < 0.001) indicating that those with a higher CS would typically exhibit lower amounts of D’ depletion during match-play regardless of the absolute magnitude of their D’. This suggests that certain players may exhibit more efficient energy utilization during performance above CS as indicated by the greater propensity to initiate HSR (r = 0.47, p = 0.015) and VHSR (r = 0.33, p = 0.010) intervals. In practical terms, this relationship implies that athletes who can maintain higher running speeds may experience less variation in their fatigue levels. The application of the D’_bal_ model in intermittent exercise protocols has proven useful for performance prediction, as demonstrated by Kirby et al. [17]. They further highlighted that D’ is highly influenced by the intensity and duration of recovery phases, therefore, incorporating D’_bal_ into real-time player load monitoring could improve the accuracy of fatigue management and decision-making, emphasizing the need for individualized, context-specific performance evaluations.

Our results also indicated that key 3MT-related metrics exhibited moderate-to-strong associations with traditional match-based metrics. More specifically, Initiate_Smax_ from the 3MT showed a strong positive correlation with Initiate_VHSR_ (r = 0.73, *p* < 0.001) and a moderate positive correlation with D_>VHSR_ (r = 0.69, *p* < 0.001)_,_ indicating that players who frequently initiated speeds above 90% of their maximal running speed from a 3MT, also tend to engage in very high-speed efforts and cover significant distances at these intensities in matches. Furthermore, D_>Smax_ showed strong positive relationships with high speed running parameters, such as Initiate_VHSR_ (r = 0.76, *p* < 0.001) and D_>VHSR_ (r = 0.72, *p* < 0.001)_,_ underscoring the plausible link between *conventional* high speed running parameters from a match and variables from a 3MT. Overall, the magnitude of several observed correlations between traditional and 3MT-based metrics indicate a fundamental link whereby the latter is more reflective of the underlying physiological connection that may ultimately better explain on-field performances. These findings are consistent with the outcomes of Kramer et al. [21], who reported a strong positive relationship between CS and maximal distance covered during an all-out sprint test (r = 0.85, *p* < 0.001).

With regards to finite energy reserves, players with a higher D’ could initiate a greater number of intervals above CS (r = 0.58, p = 0.002), indicating that the depletion of their available energy reserves during match play might be linked to more frequent intense efforts. Interestingly, players with a higher S_max_ from the 3MT were found to cover less distance at speeds exceeding 90% of their maximal sprint speed during match play (r = -0.62, *p* < 0.001), suggesting a potential area for performance enhancement (e.g., incorporate training based on the anaerobic speed reserve). It is important to remember that, given the physiological underpinnings of CS and D’, these metrics would be sensitive to training-induced changes. It is possible that players had undergone training programs focused on developing short-duration, explosive power, which enhances their ability to reach high speeds over brief intervals. On the contrary, Kramer et al. [21] noted that improved sprinting speeds enabled greater distances to be covered during an all-out sprint test. However, such training may not emphasize the capacity to sustain these speeds or maintain consistent high-intensity performance over the course of a match.

The comparison between *traditional* and *modified* player load monitoring methods revealed a high correlation (r = 0.99) but also statistically significant differences (M_diff_ = 0.05 a.u., *p* < 0.001). Although these differences may seem minor, when scaled back to the original units, they amount to about ~50 played load units, The effect size for the observed difference was moderate to large (*g* = 0.73), indicating that the difference, while statistically significant, is also practically meaningful. This suggests that even small variations in player load measurements can influence decisions regarding training intensity, recovery strategies, and in-game performance management, all of which are crucial for optimizing player performance. Further research is necessary to determine the practical significance of these findings in real-world applications.

The key advantage of the *modified* method lies in its incorporation of individualized metabolic demands, as highlighted by Thomas et al. [20]. Unlike *traditional* methods which rely on generalized high-intensity running thresholds, the *modified* approach accounts for each player’s unique physiological responses, potentially providing more accurate insights into fatigue and recovery. This allows for more tailored training and recovery strategies. Moreover, *traditional* metrics often confine player load within specific speed-based intensity zones, leading to potential oversimplifications and underestimations of perceived effort. For example, one player might predominantly accumulate load in the heavy intensity zone, while another operates more in the severe intensity zone, reflecting differences in effort and energy demands not captured in the traditional model. The *modified* method captures this variability, offering a more nuanced understanding of each player’s workload and physiological responses. CS-based metrics are more sensitive to fluctuations in player performance, as they account for individualized metabolic demands, providing a more detailed understanding of load impacts on fatigue and performance. While CS-based metrics may require more initial setup, they offer real-time feedback once integrated, enabling data-driven decisions during training and matches, such as D’_bal_, which tracks finite energy reserves. This enables coaches to implement the modified player load metric in real-time to tailor training intensity, manage fatigue, and make informed substitution decisions based on individualised data, a feature not possible with *traditional* methods based on ‘static’ thresholds. Thus, a *modified* method not only personalizes load management by identifying critical fatigue thresholds, but also enhances performance and potentially reduces injury risks during matches, as emphasized by Kirby et al. [17], although these aspects require further research.

Our research focused exclusively on evaluating external workload in relation to the CS concept, without considering comparisons to conventional internal workload measures such as rating of perceived exertion or heart rate, which should be explored in future studies. Additionally, several other limitations must be acknowledged: the study was conducted on university-level male soccer players, limiting the generalizability of the findings to different competitive levels or to female athletes. The data was collected over a limited number of matches in one season, which may not fully capture performance variations across multiple seasons or different phases of the same season. Furthermore, the study focused solely on match data, excluding training sessions, which are critical for understanding total workload and recovery. Addressing these limitations in future research would provide a more robust understanding of the CS concept in soccer. We recommend that future research further investigate these relationships across various athletic populations and contexts, focusing on the practical implementation of individualized performance metrics and load management strategies to optimize player performance in soccer.

## Conclusion

Our study underscores the importance of individualized approaches to performance analysis and load monitoring in soccer. While *traditional* methods for measuring player load are reliable, a *modified* approach’s consideration of individual metabolic demands offers a more nuanced understanding of player fatigue and recovery requirements. This adaptability has the potential to enhance player monitoring and performance strategies.
